# Silica Aerogels/Xerogels Modified with Nitrogen-Containing Groups for Heavy Metal Adsorption

**DOI:** 10.3390/molecules25122788

**Published:** 2020-06-17

**Authors:** João P. Vareda, Artur J. M. Valente, Luisa Durães

**Affiliations:** 1 University of Coimbra, CIEPQPF, Department of Chemical Engineering, Rua Sílvio Lima, 3030-790 Coimbra, Portugal; luisa@eq.uc.pt; 2 University of Coimbra, CQC, Department of Chemistry, Rua Larga, 3004-535 Coimbra, Portugal; avalente@ci.uc.pt

**Keywords:** heavy metal, adsorption, silica aerogel, amine, copper, lead, cadmium, nickel

## Abstract

Heavy metals are common inorganic pollutants found in the environment that have to be removed from wastewaters and drinking waters. In this work, silica-derived aerogels and xerogels were modified via a co-precursor method to obtain functional adsorbents for metal cations. A total of six formulations based upon four different functional precursors were prepared. The materials′ structural characterization revealed a decreased porosity and surface area on modified samples, more prominent in xerogel counterparts. Preliminary tests were conducted, and the prepared samples were also compared to activated carbon. Three samples were selected for in-depth studies. Isotherm studies revealed that the pre-selected samples remove well copper, lead, cadmium and nickel, and with similar types of interactions, following a Langmuir trend. The adsorption kinetics starts very fast and either equilibrium is reached quickly or slowly, in a two-stage process attributed to the existence of different types of active sites. Based on the previous tests, the best sample, prepared by mixing different functional co-precursors, was selected and its behavior was studied under different temperatures. For this material, the adsorption performance at 20 °C is dependent on the cation, ranging from 56 mg·g^−1^ for copper to 172 mg·g^−1^ for lead.

Academic Editor: Encarnación Ruiz Ramos

## 1. Introduction

Since their conception in 1931 by Kistler, aerogels have gained growing interest from the scientific community due to their unique outstanding properties [1,2,3]. As a result, their production methods as well as their fields of application have been diversified [1,4,5]. More recently, environmental applications of aerogels have gained traction, and come as natural fit for these high surface area, porous materials. Aerogels have been employed, for example, in carbon dioxide capture and removal of organic compounds and heavy metals [3,6,7,8,9,10,11]. Their persistence in the environment, accelerated release, associated with modern activities from human society, and high toxicity have made heavy metals priority pollutants, with some of them being restricted [7,12,13].

Different materials [14], including aerogels (silica-based, carbon, organic, other inorganic types and composites thereof) have been studied as heavy metal adsorbents by several authors [6,7,8,9,15,16,17,18,19]. These works are mostly based on featuring adequate surface groups for the adsorbent to interact with the cations in solution, namely amine groups (although thiol and carboxylic groups are also common). For silica aerogels, Štandeker [20], Pouretedal [21] and Mirzaee [22] prepared adsorbents based on thiol modification and Faghihian [23,24], Ali [25], Xiaonan [26] and Huang [27] prepared materials using different amine functional groups. Fan [28] prepared a disulfide bridged silica xerogel for the same application. The authors have also reported silica-based aerogels and xerogels containing those functional groups [29,30,31]. Despite having similar modifications, in the aforementioned studies, the adsorption capacity reported differ significantly, due to the importance of the procedure and composition of the silica backbone. 

In this work, we focus on the synthesis of silica-based aerogels and xerogels modified with different nitrogen containing groups, namely primary amines, secondary amines, urea and isocyanurate. Our goal is to assess the importance of the functional group on the usability of silica-based aerogels as heavy metal adsorbents rather than solely evaluating the fitness of one modified material. To achieve this goal, we tested non-modified and differently modified silica-based aerogels on their interaction with heavy metal cations in batch adsorption tests. 

## 2. Results and Discussion

### 2.1. Prepared Adsorbents

Table 1 presents the aerogel/xerogel systems studied in this work as adsorbents. Further information about the synthesis procedures and conditions can be found in Section 3.

**Figure 1:**
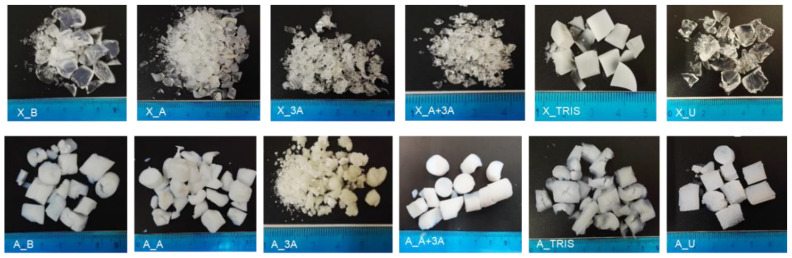
Photographs of the prepared functional aerogel and xerogel adsorbents.

Photographs from the synthesized aerogels and xerogels are depicted in Figure 1. The differences observed from the analysis of different samples are mainly due to the aerogel and xerogel counterparts. All xerogels have shrunk considerably and feature a semi-translucid, glassy aspect. Samples X_B, X_TRIS and X_U did not break into small fragments, retaining some monolithic structure. Formulation 3A generated aerogels that differ significantly from the remaining ones. In fact, these were the most difficult gels to dry using supercritical carbon dioxide (scCO_2_). The remaining aerogels are very similar and completely white.

It was observed that the introduction of amine functional groups on the solid matrix hindered the solubility of the liquid phase of the gel on scCO_2_. This was attributed to a higher retention of water (weakly soluble in scCO_2_ at low temperatures [32]) via hydrogen bonding. This situation has been overcome by employing long gel washing steps.

### 2.2. Adsorbents Characterization

The visual differences revealed by Figure 1 are further evidenced by the physical properties reported in Table 2. In a general way, aerogels are one order of magnitude lighter and more porous than xerogels. It was not possible to report all properties of samples X_3A and X_U, in particular those related with surface area and porosity, due to the lack of reliability of the obtained values in these cases. The contraction of aerogels during nitrogen adsorption [33] could have inhibited the exposure of the pores, in particular for these samples of complex and closed structure. Furthermore, these samples could be mainly microporous and with low surface areas, and their proper assessment becomes limited given the test conditions [34,35]. The difficulty in obtaining the porosity value in sample X_U comes from the limited way of approximating bulk density, being overestimated.

The presence of a primary amine or isocyanurate groups in samples A_A/A_A+3A and A_TRIS respectively, did not alter the bulk density, porosity and pore volume greatly in comparison with sample A_B. On the other hand, the introduction of any functional group caused the specific surface area to decrease, due to an increase in the average pore size. This is associated with the catalytic effect of amine groups in sol-gel chemistry [36]. Aerogel samples A_3A and A_U differ significantly, in regard to the properties presented in Table 2, from the remainder samples. As mentioned previously, sample A_3A did not retain its porous structure during drying, hence the reduced porosity observed. Sample A_U is denser and less porous than the majority of modified aerogel samples. This could be due to the urea groups that also catalyze sol-gel reactions and can generate hydrogen bonds through the nitrogen or oxygen atom of these groups.

**Figure 2:**
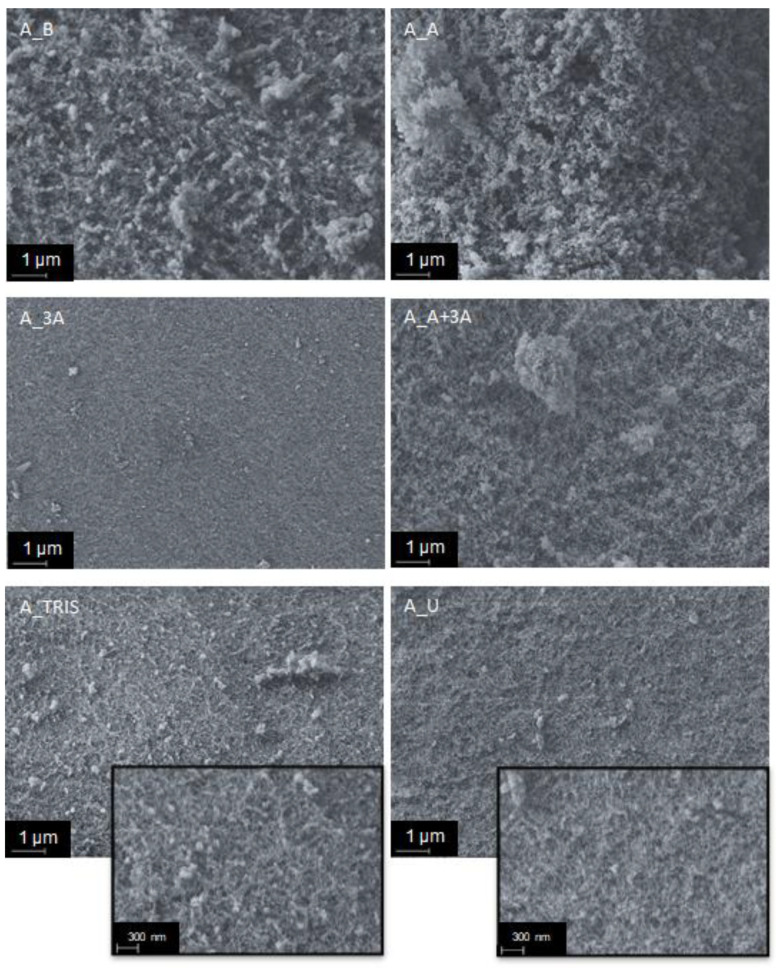
Morphology of the prepared aerogel samples (10,000× magnification) and details for samples A_TRIS and A_U (30,000× magnification).

Figure 2 shows scanning electron micrographs of the different aerogels. The samples can be divided into two contrasting groups: samples A_B, A_A and A_A+3A are clearly porous, while A_3A, A_TRIS and A_U show a much more closed and featureless structure, being A_3A the most compact. Samples of the first group feature the expected microstructure for silica aerogels. They show a porous matrix formed by aggregated secondary silica particles of very small size. The remaining samples are mainly microporous, and their microstructure is similar to that of modified xerogels of an earlier work [29].

The introduction of the propylamine groups in the silica backbone (sample A_A) seemed to have a small effect in the microstructure of the aerogels (Table 2, Figure 2), when compared to the reference sample. However, this is not the case with formulation 3A that includes the functional groups containing three amines (one primary and two secondary), which affected significantly the properties of this formulation. This can be possibly explained by the size of the organic group in the silane, that may induce less ramified structures, and/or due to the presence of three amine groups, which may extensively catalyze the condensation reactions. Formulation A+3A shows properties in-between the former two, as expected. Sample A_TRIS has high surface area and pore volume, not that different from sample A_A (Table 2), but the micrograph reveals a structure that seems less porous than the latter. Nevertheless, a great number of small pores, that seem uniform in size, are visible in the surface of the sample (see the inset in Figure 2). With formulation U, the propylurea groups led to the second most closed structure (less porous), right after sample A_3A, in agreement with results of Table 2.

**Figure 3:**
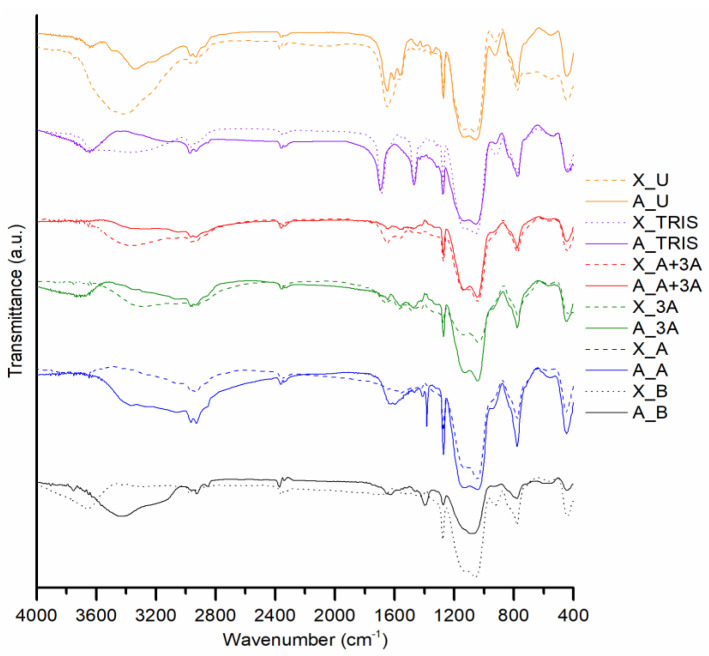
FTIR spectra for different xerogels and aerogels.

The chemical composition of the adsorbent samples was studied with infrared spectroscopy—Figure 3, and with elemental analysis—Table 3.

The samples′ FTIR spectra presented in Figure 3 reveal the typical silica matrix expected for silica aerogels and xerogels [37], through the bands at ~443, 554, 778, 932 (shoulder), 1047 and 1130 cm^−1^, attributed to bending vibrations of siloxane bonds, SiO_2_ defects, symmetric stretching of siloxane bonds, stretching of silanol bonds and the two vibrational modes of asymmetric stretching of siloxane bonds, respectively. All formulations have very similar spectra, as they are based on nearly similar silica backbone and the different functional groups contain the same covalent bonds, in most cases. The main bands related to the functional groups are visible at ~720 (shoulder), 831 (shoulder), 1273–1470, 1560–1645, 1668 and 2850–2975 cm^−1^, that are ascribed to the deformation of methylene groups, stretching of silicon-carbon bonds, bending of methylene and methyl groups, bending of primary and secondary amines, stretching of carbonyl bonds and stretching of methylene and methyl groups. Despite amine and hydroxyl bonds generating bands in the same region of the spectra, the 1500–1650 cm^−1^ region of the spectra is different between formulation B and the remaining formulations, evidencing the presence of N-containing groups in the functionalized samples. The region between 4000 and 3100 cm^−1^ of the spectra varies between formulations due to the same reason.

For comparison with the experimental results from elemental analysis, different theoretical hypotheses were considered for the condensation of the precursors. These theoretical scenarios assume that the hydrolysis of precursor molecules is complete, and all precursor molecules react to form the gel backbone. The different scenarios vary in the number of hydroxyl groups that are left unreacted, per precursor molecule, hence ranging from a complete condensation to an incomplete condensation where two hydroxyl groups are left unreacted in each molecule.

The elemental analysis results reveal in some cases (samples A_B, A_A, X_TRIS, A_TRIS and A_U) that the content of carbon is greater than that predicted by the theoretical scenarios. This can be attributed to the fact that hydrolysis was not complete (hence methoxy or ethoxy groups still exist in the silica backbone) or to the possible heterogeneity of the sample, with the quantification occurring in portions where some precursors are more prominent. In fact, Itagaki et al. [38] showed that the precursors tend to condense with similar species in some situations, and so phase segregation of condensed precursor species can occur. Analyzing the carbon content, most samples are between the values of complete condensation (CC) and incomplete condensation with one hydroxyl group remaining per precursor molecule (IC 1OH). This is in general corroborated by the results of N content. However, the analysis of the N content of formulation B reveals that, despite being washed, aerogels still have ammonia catalyst residues (as already hypothesized in the FTIR discussion), contributing to some uncertainty. For the case of samples of formulations 3A and A+3A, the nitrogen content seems to suggest the more incomplete condensation scenario, which is not in agreement with the carbon result.

### 2.3. Preliminary Adsorption Tests

Preliminary tests were conducted with every sample from Table 1 in order to assess which ones were the most viable for the removal of different heavy metals. The results from this screening, based on two replicates, are presented in Table 4. A concentration of 50 mg·L^−1^ of the cations was used in these tests.

The obtained results show that activated carbon, considered as a standard adsorbent, is not a viable material for the removal of the tested cations, showing removal efficiencies below 20% (Table 4). On the other hand, the materials of formulation B do not interact well with the cations, apart from lead which has considerable removal efficiencies at the tested concentration. In fact, lead is fairly removed by all materials, compared to the other cations, because lead has the lowest charge density of the four, and thus has the less stable hydration sphere, being more labile [39,40].The removal of lead could be due to silanol groups, featured in all silica-based adsorbents or to non-specific processes like electrostatic interactions with the negatively charged [41,42] silica surface. Despite having functional groups containing electron donor atoms, i.e., Lewis bases, the results obtained with aerogels and xerogels of formulations TRIS and U are nearly similar to those of formulation B. In fact, these three formulations do not show good performance (except for lead), even when compared with activated carbon. This is a consequence of the hindering of active surface sites, in modified samples, towards the metal ions. This can be justified by the limitations in the adsorbent’s microstructure/physical properties (formulation U) or by the absence of functional Lewis base groups at the surface of the adsorbent (formulation TRIS). As the isocyanurate ring is enclosed by three silicon atoms with sol-gel reactive groups, it is not so available on the surface of silica particles—contrary to what happens with the remaining precursors. The materials from formulations A, 3A and A+3A show better adsorption performances. Samples X_3A and A_3A are analogous in terms of structural properties (Table 2) and adsorption performance (Table 4). Samples A_A and A_A+3A perform better and more consistently across the four tested cations when compared to their xerogel counterparts and the remaining formulations. While for copper and lead, the lower surface area and porosity of the xerogels does not inhibit the adsorption process (suggesting that the active sites are accessible in the xerogels), this is not the case for cadmium and nickel. Because all cations are fairly similar in size, the surface heterogeneity or a different adsorption mechanism for the latter cations may explain these results.

The initial screening of the adsorption performance of the samples allows us to conclude that A_A and A_A+3A are good adsorption candidates for the studied heavy metals. X_3A also performs fairly well and was also selected for being easier to obtain than its aerogel counterpart.

### 2.4. Effect of pH and Adsorbent Dose

**Figure 4:**
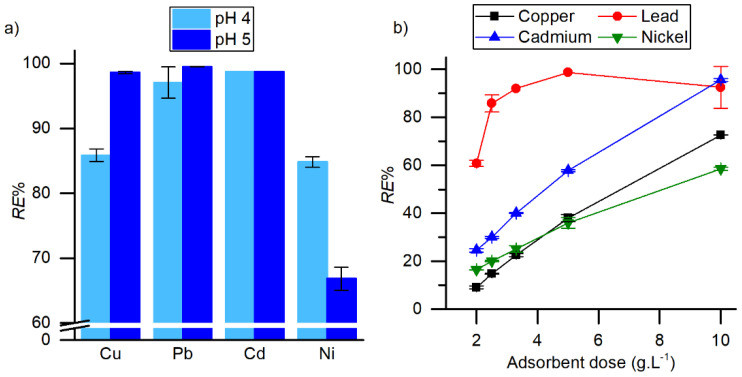
The effect of (**a**) initial solution pH at *C*_0_ = 50 mg·L^−1^ and (**b**) adsorbent dose at *C*_0_ = 500 mg·L^−1^ on heavy metals removal by aerogel A_A.

The effect of pH and adsorbent dose on the adsorption performance was studied using sample A_A. The results are presented in Figure 4. To evaluate the effect of the initial solution′s pH, a starting concentration of 50 mg·L^−1^ of cation and 2 g·L^−1^ of adsorbent were used. The results from Figure 4a show that only copper adsorption was significantly decreased by the decrease in pH to 4. Nickel shows the opposite trend, while for lead and cadmium the pH variation effect was not significant, and the detection limit was sometimes achieved. pH of 4 was chosen to guarantee that no metal hydroxides are formed. At pH 4 and with a starting concentration of 500 mg·L^−1^, the removal of the metal ions was quantified as a function of the adsorbent dose (Figure 4b). As expected, the greater the mass of adsorbent the greater the mass of pollutant that can be removed. At the highest mass of adsorbent very significant amounts of cations were removed; in particular it can be observed an almost complete removal of lead and cadmium. Although at a high pollutant concentration, high removals are only expected with greater adsorbent doses, a small dose of 2 g·L^−1^ was selected in this work to obtain adsorption isotherms and kinetic curves since wastewaters typically feature low concentrations of these pollutants due to high dilution.

### 2.5. Adsorption Kinetics

The adsorption kinetic models used in this study, in their integrated forms, are presented in Equations (1)–(3). In these, *q* is the sorbate uptake (mg.g^-1^) at equilibrium (*q*_e_) or at a given time *t* (*q*_t_), being *t* in hours. The quality of the fits was assessed using Akaike Information Criterion (AIC) and Bayesian Information Criterion (BIC) [43]. The pseudo-first order or Lagergren model [44] is presented in Equation (1) and *k*_1_ is the first order rate constant (h^−1^). The pseudo-second order or Ho and McKay model [45], Equation (2), is the most common for heavy metals [6]. The pseudo-second order adsorption rate constant is represented by *k*_2_ (g·mg^−1^·h^−1^). The double exponential model (Equation (3)) [46,47] describes a two-step kinetic adsorption process, where the adsorption starts with a fast step (step 1) and then proceeds slowly towards equilibrium (step 2). In this equation, *K*_D_ (h^−1^) is the adsorption rate constant (in general, related with the diffusivity), *D_i_* (mg·L^−1^) represents the maximum adsorbed amount at step *i,* and *c* is the concentration (g·L^−1^) of adsorbent in the test:

(1) $$q_{t}{=q}_{e}\left({1-e}^{{-tk}_{1}}\right)$$

(2) $$q_{t}=\frac{q_{e}^{2}k_{2}t}{q_{e}k_{2}t+1\;}$$

(3) $$q_{t}=q_{e}-\frac{D_{1}}{c}e^{-K_{D1}t}-\frac{D_{2}}{c}e^{-K_{D2}t}$$

**Figure 5:**
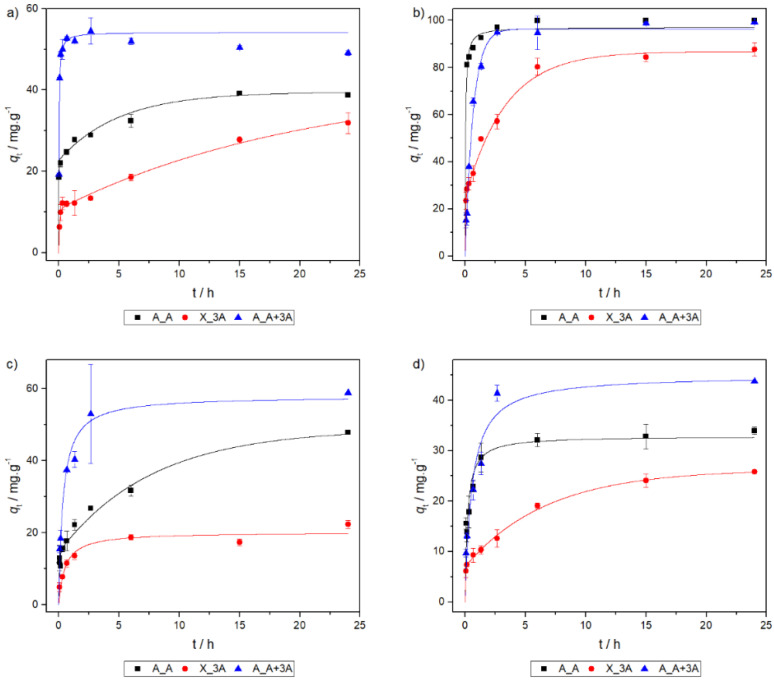
Sorption kinetics for (**a**) copper, (**b**) lead, (**c**) cadmium and (**d**) nickel.

Adsorption kinetics were studied for adsorbents A_A, X_3A and A_A+3A. Kinetic models were fitted to data from kinetic tests and the results are shown in Figure 5 and Table 5. For the sake of simplicity, only the best model is represented in the graphs. The selection of the kinetic models was not solely based on Akaike′s and Bayesian Information Criteria, as these are often in disagreement: BIC suggests that the double exponential model (DEM) is usually the best model while AIC penalizes this model severely for having too much parameters for the number of data points, even when it is clearly the most adequate (see Figure 5). The kinetic tests reveal two different situations: the adsorption process occurs in two-steps (DEM model) or in one, being controlled by the rate of sorption phenomena at the adsorbent surface (pseudo-second order (PS2) model). This result was also observed in other functionalized silica materials [47]. The DEM model generally assumes that the steps are diffusion limited but it has also been shown that this model can describe adsorbents with two types of active sites present [47], situation in which the process is not limited by diffusion.

The DEM model is mostly observed with adsorbent X_3A, which has little porosity and features two different kinds of active sites: primary and secondary amines. However, it is also the best model to describe the kinetics of A_A with copper and cadmium. In these situations, a clear two-step mechanism is observed. By looking for a mechanism, it can be hypothesized that the slow step can be attributed to the interaction of the metal ions with other groups on the adsorbent, such as silanol, whilst the fast one is due to the interactions with the primary amine groups. For the situations under study, the adsorption steps from the DEM model are not a result of diffusional limitations of the adsorption process: the experimental points are well described by the pseudo-second order model in the rapid step (Appendix A), evidencing that this step is linked to a chemical reaction at the surface of the adsorbent, hence, the process is due to the existence of two types of active sites.

The interactions of A_A+3A with lead seem to be controlled by the bulk concentration as evidenced by the pseudo-first order model. For the remaining situations, the sorption kinetics is limited by the surface reactions, meaning that chemisorption is most likely occurring (model PS2). It is worth noticing that adsorbent A_A+3A, which also features different types of active sites, does not have its behavior modeled by the DEM equation. Furthermore, it behaves differently than A_A meaning that the secondary amine groups are still somewhat accessible. It is possible that due to its extensive porous structure, diffusion occurs quickly and the functional groups are very accessible, interacting fast and in one step.

### 2.6. Adsorption Isotherms

Relevant adsorption models [6], namely Langmuir and Freundlich isotherm models, were fitted to the data using nonlinear algorithms. The quality of the fits was also assessed by using Akaike and Bayesian criteria.

In the Langmuir model, Equation (4), *K*_L_ is the Langmuir constant (L·mg^−1^) and *q*_max_ represents the monolayer adsorption capacity of the adsorbent (mg·g^−1^). The Freundlich model, Equation (5), also has two parameters: the Freundlich constant, *K*_F_ ((mg·g^−1^) (L·mg^−1^)^1/n^_F_) and the heterogeneity factor 1/*n*_F_ [6].

(4) $$q_{e}=\frac{q_{\max}K_{L}C_{e}}{{1+K}_{L}C_{e}}$$

(5) $$q_{e}{=K}_{F}C_{e}{}^{\frac{1}{n_{F}}},$$

**Figure 6:**
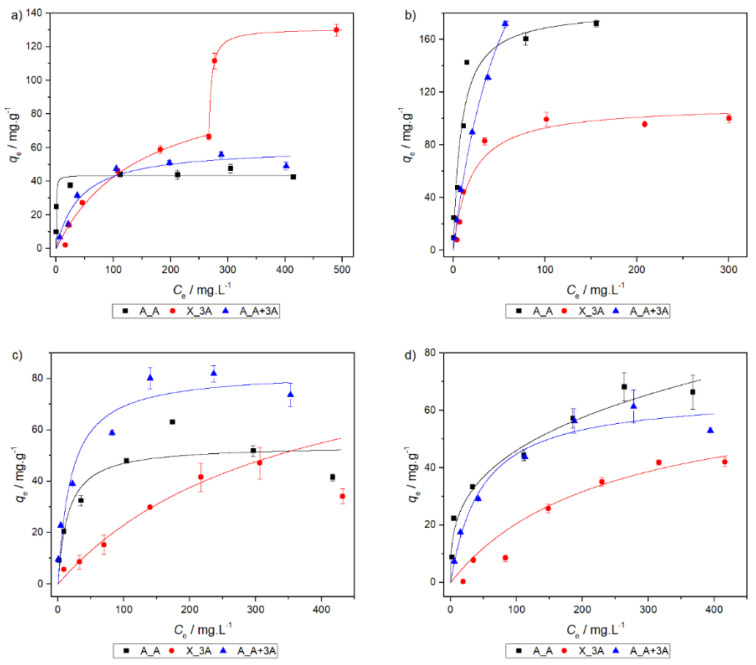
Sorption isotherms for (**a**) copper, (**b**) lead, (**c**) cadmium and (**d**) nickel.

Adsorption isotherm curves for adsorbents A_A, X_3A and A_A+3A are presented in Figure 6. For clarity, only the best fitting model is represented in the graphs. The fit parameters are compiled in Table 6. Figure 6 and Table 6 show that the Langmuir equation is the best model in most of the cases. This suggests that the surface of the adsorbents can be considered homogeneous and that the adsorption process is due to the interaction at active surface sites—the amine functional groups. This result is expected [30,31] and corroborates the predictions from the hard and soft acids and bases theory. Notable exceptions to this trend are obtained for the systems A_A with nickel and X_3A with copper. In the first situation the model that best fits to the data is the Freundlich model, suggesting that the interactions with this cation occur differently than in other situations, and the adsorbent surface cannot be considered homogeneous. In the second situation, an L4 isotherm is observed [48], which is characterized by the existence of two plateaus due to the development of a new surface where adsorption can occur. Thus, the second plateau indicates the saturation of the new surface and the complete saturation of the adsorbent. This could be due to the reorientation of previously sorbed species, leading to partial uncovering of the adsorbent surface [48]. The isotherm for each plateau is given in Table 6. It is suggested that the Langmuir model fits each plateau better.

Although the different adsorbents have similar behaviors, adsorbent A_A+3A is found to be the best out of the three tested in depth, for the majority of the situations.

### 2.7. Adsorption Thermodynamics

**Figure 7:**
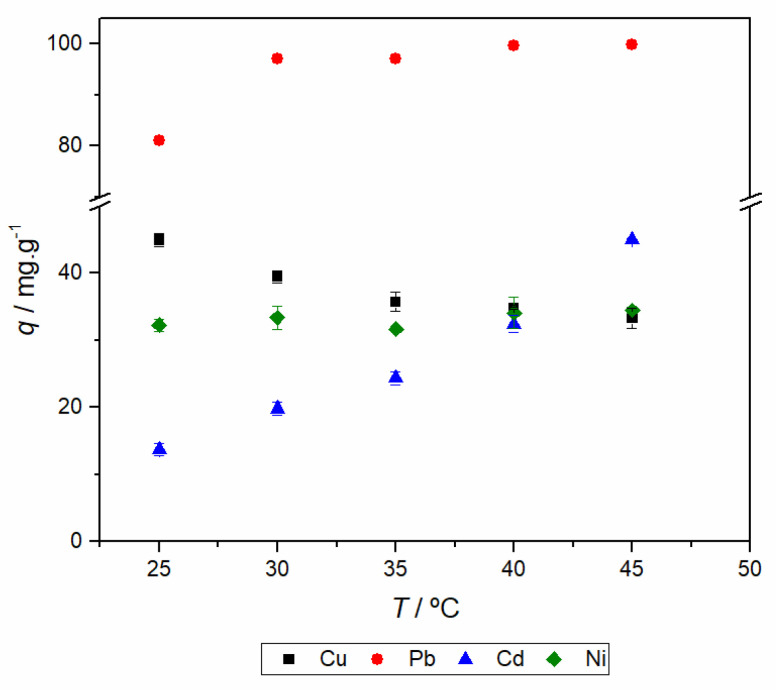
Effect of temperature on the adsorption performance of A_A+3A.

The adsorption capability of aerogel A_A+3A was studied with varying temperature, and its results are summarized in Table 7 and Figure 7. The standard Gibbs energy, enthalpy and entropy changes of adsorption were estimated as follows. In this work, only one adsorbate concentration was tested at different temperatures, consequently, the equilibrium constant at each temperature was estimated by *K*_d_ (L·g^−1^) (Equation (6)), which was used to calculate the standard adsorption Gibbs free energy with Gibbs equation (Equation (7)). The standard adsorption entropy and enthalpy variations were estimated by linear regression using a modified van’t Hoff’s equation (Equation (8)):

(6) $$K_{d}=\frac{q_{e}}{C_{e}},$$

(7) $$\Delta G^{0}=-RT\ln K_{d},$$

(8) $$\ln K_{d}=\frac{\Delta S^{0}}{R}-\frac{\Delta H^{0}}{RT},$$

By the analysis of the standard Gibbs energy change, for all metal ions, the adsorption process is spontaneous. However, although for lead and cadmium the adsorption stability becomes higher by increasing the temperature, for copper and nickel only slightly variations of the Gibbs energy are observed. This can be justified by an athermic adsorption process (*i.e.*, *K*_d_ is independent on temperature as observed for nickel) or by an entropy-enthalpy compensating effect [49]. Another possible justification, by comparing the thermodynamic functions of nickel(II) and copper(II), is related with the configuration of hexahydrate complexes; while the Ni(H_2_O)_6_^2+^ has a symmetrical octahedron configuration, the hexacoordinated copper(II) shows a Jahn-Teller distortion; consequently the shortest distance Cu-O(H_2_O) is smaller than the distance Ni-O(H_2_O) (1.96 and 2.055 Å, respectively) and thus the ability of copper to interact with the adsorbent is higher [50]. Looking to the thermodynamic function values shown in Table 7, it can be concluded that the enthalpy change of sorption increases in the order Cu(II) < Cd(II) < Pb(II). This is in close agreement with the effect of the metal ion radii (*r*_i_): 0.73, 0.95 and 1.19 Å, respectively [50]; *i.e.,* the interaction adsorbate-adsorbent is higher by increasing the charge density of metal ions. This also indicates that the interactions are of electrostatic nature. However, for Cd(II) and Pb(II) the sorption is characterized by an endothermic process and only for Cu(II) the sorption is slightly exothermic, suggesting that the sorption interaction between metal ions and the adsorbent is weaker [51,52]. This also explains the dependence of the Gibbs energy change of copper and nickel (r_i_ = 0.69 Å [50]) with the temperature, since the stability of hydration shell is higher and, consequently, these metal ions are the less labile [39]. This is in line with the entropy variation values for the different metal ions; in fact, the water loss from the hydration sphere of more labile metal ions plays a major role in driving the adsorption process [39].

## 3. Materials and Methods

### 3.1. Materials

Methyltriethoxysilane (MTES, ≥99%, Sigma-Aldrich, Darmstadt, Germany), tetraethylorthosilicate (TEOS, 98%, Sigma-Aldrich, Darmstadt, Germany), (3-aminopropyl)trimethoxysilane (APTMS, ≥97%, Sigma-Aldrich, Darmstadt, Germany), N1-(3-trimethoxysilylpropyl)diethylenetriamine (AAAPTMS, technical grade, Sigma-Aldrich, Darmstadt, Germany), 1-[3-(trimethoxysilyl)propyl]urea (UPTMS, 97%, Sigma-Aldrich, Darmstadt, Germany) and Tris[3 -(trimethoxysilyl)propyl] isocyanurate (TTMSI, >95.0%, Fluorochem, Derbyshire, United Kingdom) were used as silica sources. Methanol (99.8%, VWR International, Alfragide, Portugal) and ethanol (EtOH, ≥99.8%, Fisher, Porto Salvo, Portugal), anhydrous oxalic acid (≥99%, Sigma-Aldrich, Darmstadt, Germany), ammonium hydroxide (25% NH_3_ in H_2_O, Sigma-Aldrich, Darmstadt, Germany) were used as solvents and catalysts for the sol-gel chemistry. Heavy metal solutions were prepared using copper(II) nitrate hemipentahydrate (*p.a.*, Chem-Lab, Zedelgem, Belgium), lead(II) nitrate (≥99.0%, Sigma-Aldrich, Darmstadt, Germany), cadmium nitrate tetrahydrate (≥99.0%, Sigma-Aldrich, Darmstadt, Germany), nickel(II) nitrate hexahydrate (crystals, Sigma-Aldrich, Darmstadt, Germany). High purity water was used whenever needed. Nitric acid (65%, Fisher) was used to adjust the solutions pH.

### 3.2. Synthesis of Silica Aerogels and Xerogels

Six different formulations were prepared, mixing different co-precursors. Each of them is summarized in Table 1. Formulations A and B were introduced by the authors in an earlier work [31].

Organically modified silica aerogels and xerogels were prepared using the procedures previously reported by the authors [29,30,31]. In sum, the precursors are mixed in methanol and, then an aqueous oxalic acid solution (1 M) is added to enhance the hydrolysis of precursors. This solution is maintained in an oven at 27 °C during 24 h. Subsequently, an aqueous ammonium hydroxide solution is added to the previous mixture and the sol is left to gel and age. Gelation time varies from a few minutes to a couple of hours. The Si:solvent:acid water:basic water ratios were kept at 1:12:4:4. To prepare aerogels, gelation and aging occurred in cylindrical polypropylene molds and the samples were washed with hot ethanol followed by supercritical drying with CO_2_. To prepare xerogels, the alcogels were dried in an oven at 60 °C for three days. The condensation/gelation conditions were adjusted for obtaining cohesive gels. These conditions are summarized in Table 8, along with the registered gel times.

### 3.3. Adsorption Tests

The adsorption experiments followed the procedures used previously [29,30,31]. The prepared aerogels and xerogels were milled and sieved to obtain a powder with a particle size distribution of 75 to 250 μm. The powdered adsorbent and the cation solution are mixed in a test flask, that is shaken in a rotational stirrer at 16 rpm (REAX 20, Heidolph Instruments, Schwabach, Germany) or in a ProBlot 6 Hybridization Oven (Labnet International, Inc, Edison, NJ, USA) for the case of the thermodynamic tests. When the test ends, the solution is filtered, and the concentration of the filtrate is determined.

Initial screening of adsorbents was performed at pH5, with an adsorbent dose of 2 g·L^−1^, at 20 °C and 24 h of contact time. Commercial activated carbon (activated charcoal suitable for cell culture, Sigma-Aldrich) was also tested for comparison purposes. Afterwards, the effect of different test parameters on the adsorption performance was studied: initial adsorbate solution pH was varied between 4 and 5 and adsorbent concentration ranged from 2 to 10 g·L^−1^. pH 4 and 2 g·L^−1^ of adsorbent concentration were selected for the subsequent experiments.

Batch equilibrium tests were performed by changing the adsorbate concentration from 20 to 500 mg·L^−1^, and conducted for 24 h. For batch kinetic tests, contact times ranged from 1 min to 24 h, with an adsorbate concentration of 200 mg·L^−1^. These tests were conducted at 20 °C. Thermodynamic tests were conducted with the same 200 mg·L^−1^ adsorbate concentration, and with a temperature range of 25–45 °C.

From the initial screening, A_A adsorbent was selected to study the effect of test parameters and three adsorbents were selected to study in depth with batch isotherm and kinetic tests (A_A, X_3A, A_A+3A). The influence of the adsorption temperature was studied with the best adsorbent (A_A+3A).

Adsorption capacity (*q*_t_ or *q*_e_ if equilibrium is reached, mg·g^−1^) and the removal efficiency (*RE*, %) were calculated from the initial (*C*_0_, mg L^−1^) and final concentrations (*C*_t_ or *C*_e_, mg·L^−1^), adsorbent mass (*m*, g) and solution volume (*V*, L) according to Equations (9) and (10), respectively:

(9) $$q_{e}=\frac{V\left(C_{0}-C_{e}\right)}{m},$$

(10) $$RE=\frac{100\left(C_{0}-C_{e}\right)}{C_{0}},$$

### 3.4. Characterization

Bulk density of the adsorbents was obtained by weighting portions of sample and assessing its volume: by measuring geometrically regular pieces on the three axes, or by liquid displacement in the case of some xerogels, due to randomness of particle shapes. Adsorbent porosity was calculated using the bulk and skeletal densities, the latter obtained with powdered samples by He pycnometry (Accupyc 1330, Micrometrics, Norcross, GA, USA) [53]. Nitrogen adsorption (ASAP 2000, Micrometrics) was used to obtain the Brunauer Emmett Teller (BET) specific surface area. Pore volume and average pore size were calculated in accordance to a previous work [53]. Scanning electron microscopy (SEM) was used to observe the samples’ microstructure (Merlin Compact/VPCompact FESEM, Zeiss, Carl Zeiss Microscopy GmbH, Jena, Germany)

Infrared spectra (FT/IR 4200, Jasco, Easton, USA) were obtained with KBr pellets of each sample, with a wavenumber range of 4000 to 400 cm^−1^, 128 scans and a resolution of 4 cm^−1^. C, H and N content of powdered samples was determined by elemental analysis (EA 1108 CHNS-O, Fisons, Ipswich, United Kingdom). Heavy metal concentration in solution was determined by Flame atomic absorption spectroscopy with an acetylene-air flame (939 AAS, Unicam, Cambridge, UK).

## 4. Conclusions

Different co-precursors with Lewis base functional groups based on amines were used to produce silica-derived aerogels and xerogels that can be applied as heavy metal adsorbents, namely for copper, lead, cadmium and nickel. The following conclusions were drawn from this study:

(1) The presence of the N-containing functional groups in the silica backbone impacted the microstructure and the properties of the materials significantly, reducing surface areas and porosity.
(2) The drying conditions, namely at ambient pressure or in supercritical conditions, strongly influenced the materials structure, with aerogels retaining appreciable porosity and surface area and xerogels being more densified materials.
(3) The aforementioned properties affect the adsorption performance and, in general, adsorbents with low porosity were not good adsorbents.
(4) In terms of functional groups, materials derived from formulations TRIS and U were not good adsorbents for the tested cations; for the remaining formulations, with the exception of 3A, aerogels remove more cation than their xerogel counterpart, in agreement with the more extensive porosity of aerogels.
(5) It was found that pH4 is more favorable for the adsorption of the studied metals, in agreement with their speciation diagrams.
(6) The in-depth study of the best samples (A_A, X_3A and A_A+3A) revealed that the Langmuir trend is verified in almost all cases and that the kinetic process can either reach equilibrium very fast or in a two-stage process depending on the adsorbate-adsorbent pair. These two stages may be attributed to the presence of two types of active sites in some of the materials.
(7) The isotherm and kinetic models suggest, in most situations, that the prepared adsorbents removed the cations by chemisorption.
(8) Thermodynamic tests with the A_A+3A adsorbent reveal the spontaneous nature of the adsorption process, being observed that it is exothermic for copper, endothermic for lead and cadmium and athermic for nickel.

## Figures and Tables

**Table 1:** Aerogel/xerogel functional groups and samples nomenclature.

Functional Groups	Molar Composition (%) of the Precursor System ^(a)^	Xerogel	Aerogel
Ref. material (without N-containing groups)	62.5%MTES/37.5%TEOS	X_B	X_B
3-Aminopropyl	50%MTES/30%TEOS/20%APTMS	X_A	A_A
Propyl diethylenetriamine	50%MTES/30%TEOS/20%AAAPTMS	X_3A	A_3A
3-Aminopropyl + propyl diethylenetriamine	50%MTES/30%TEOS/10%APTMS/10%AAAPTMS	X_A+3A	A_A+3A
Propyl isocyanurate	50%MTES/30%TEOS/20%TTMSI	X_TRIS	A_TRIS
Propyl urea	50%MTES/30%TEOS/20%UPTMS	X_U	A_U

^(a)^—APTMS: (3-aminopropyl)trimethoxysilane; AAAPTMS: N1-(3-trimethoxysilylpropyl)diethylene triamine; TTMSI: tris[3-(trimethoxysilyl)propyl]isocyanurate; UPTMS: 1-[3-(trimethoxysilyl)propyl]-urea.

**Table 2:** Structural properties of the prepared functional aerogel and xerogel adsorbents.

Formulation		Bulk Density ^(a)^/g cm^−3^	Porosity/%	*S*_BET_/m^2^ g^−1^	*V*_pore_/cm^3^ g^−1^	*D*_pore_/nm
B	Xerogel [31]	1.07	24	761	0.22	1
	Aerogel	0.141	90	1006	6.38	25
A	Xerogel [31]	1.41	3	28	0.02	4
	Aerogel	0.134	87	573	6.48	45
3A	Xerogel	1.30	7	(b)	0.05	-
	Aerogel	0.737	48	14	0.64	182
A+3A	Xerogel	1.12	23	3	0.21	268
	Aerogel	0.191	86	256	4.49	70
TRIS	Xerogel	1.14	16	634	0.14	1
	Aerogel	0.132	88	451	6.67	59
U	Xerogel	1.42	(c)	(b)	(c)	-
	Aerogel	0.430	67	398	1.56	16

^(a)^—Values for some xerogels were obtained with liquid displacement and should be considered indicative. (b) Non-reliable result from nitrogen adsorption. (c) Residual porosity, since the skeletal and bulk densities show similar values.

**Table 3:** Chemical composition of the studied samples.

Formulation	Sample/Hypothesis ^(a)^	wt% C	wt% H	wt% N
B	Exp. Xerogel [31]	11.87	3.52	0.58
	Exp. Aerogel	15.41	4.05	0.85
	CC	11.64	2.93	-
	IC 1OH	10.22	3.94	-
	IC 2OH	9.10	4.73	-
A	Exp. Xerogel [31]	15.31	4.47	3.30
	Exp. Aerogel	19.62	4.87	3.71
	CC	17.95	4.24	3.81
	IC 1OH	15.99	5.00	3.39
	IC 2OH	14.42	5.61	3.06
3A	Exp. Xerogel	22.16	5.94	7.91
	Exp. Aerogel	22.07	5.60	7.14
	CC	25.12	5.66	9.25
	IC 1OH	22.85	6.16	8.42
	IC 2OH	20.96	6.57	7.72
A+3A	Exp. Xerogel	19.05	5.53	6.00
	Exp. Aerogel	20.98	5.14	5.52
	CC	21.91	5.03	6.81
	IC 1OH	19.75	5.63	6.14
	IC 2OH	17.97	6.13	5.59
TRIS	Exp. Xerogel	20.40	4.11	3.83
	Exp. Aerogel	21.95	4.37	3.75
	CC	19.81	3.45	3.55
	IC 1OH	18.02	4.15	3.23
	IC 2OH	16.12	4.89	2.89
U	Exp. Xerogel	17.06	4.38	5.60
	Exp. Aerogel	21.41	4.86	6.45
	CC	18.99	4.05	6.81
	IC 1OH	17.11	4.75	6.14
	IC 2OH	15.58	5.33	5.59

^(a)^ Exp—Experimental values. CC—Complete condensation. IC 1OH—Incomplete condensation where one hydroxyl group is left unreacted per precursor molecule. IC 2OH—Incomplete condensation where two hydroxyl groups are left unreacted per precursor molecule.

**Table 4:** Heavy metal removal efficiencies, in percentage, for different adsorbents. *C*_0_ = 50 mg·L^−1^, pH 5, 20 °C, 24 h.

Sample	Copper Removal (%)	Lead Removal (%)	Cadmium Removal (%)	Nickel Removal (%)
X_B	8.8	44.5	0.9	3.3
A_B	8.7	64.2	(a)	2.3
X_A	12.9	61.0	5.6	4.2
A_A	98.6	99.5	98.8	66.8
X_3A	59.0	98.9	6.05	6.1
A_3A	33.4	96.9	10.5	2.5
X_A+3A	64.7	77.7	26.6	21.8
A_A+3A	59.4	93.0	95.0	64.0
X_TRIS	10.1	34.0	(a)	(a)
A_TRIS	8.6	51.9	3.5	(a)
X_U	5.2	38.1	1.8	(a)
A_U	4.4	39.3	3.0	(a)
AC	21.7	18.6	9.4	2.8

(a)—No removal was observed.

**Table 5:** Adsorption model and fit parameters for sorption kinetics.

	Pseudo-First Order (PS1)				Pseudo-Second Order (PS2)				Double-Exponential Model (DEM)							Exp. *q*_e_ (mg g^−1^)	Preferred Model
	*k*_1_ (h^−1^)	*q*_e_ (mg g^−1^)	AIC ^(a)^	BIC ^(b)^	*k*_2_ × 10^3^ (g mg^−1^ h^−1^)	*q*_e_ (mg g^−1^)	AIC ^(a)^	BIC ^(b)^	*D*_1_ (mg L^−1^)	*K*_D1_ (h^−1^)	*D*_2_ (mg L^−1^)	*K*_D2_ (h^−1^)	*q*_e_ (mg g^−1^)	AIC ^(a)^	BIC ^(b)^		
A_A Cu	27.6	30.9	42.7	38.5	1866	31.5	39.5	35.3	45.3	102	33.6	0.18	39.5	51.2	10.3	44.0	DEM
X_3A Cu	0.43	26.8	43.5	40.4	36.3	27.2	39.8	36.7	21.7	11.8	63.2	4.8E-2	42.3	31.6	5.4	46.3	DEM
A_A+3A Cu	23.8	517	19.2	15.0	713	54.2	16.3	12.1	87.2	32.5	18.3	4.1	52.9	49.1	8.3	47.5	PS2
A_A Pb	11.2	93.3	34.5	26.4	261	97.1	26.3	18.1	161	23.4	39.1	0.73	100.0	68.7	-14.8	94.3	PS2
X_3A Pb	0.87	80.4	56.8	53.7	16.1	85.9	50.9	47.3	124	0.32	49.3	26.8	86.8	52.6	26.4	82.7	DEM
A_A+3A Pb	1.5	96.4	28.0	23.8	18.4	106.4	37.7	33.5	191	1.5	2.2	400	96.5	71.1	30.2	89.6	PS1
A_A Cd	0.93	36.9	44.5	40.3	29.0	41.5	40.1	35.9	28.6	16.0	68.7	0.14	48.9	64.0	23.2	47.9	DEM
X_3A Cd	1.3	19.2	22.2	16.4	96.4	20.2	18.0	12.3	1.1E5	4.8E-6	28.3	1.8	5.3E4	99.7	16.2	30.0	PS2
A_A+3A Cd	1.9	53.0	36.8	28.6	48.1	58.0	28.5	20.3	68.6	0.56	48.7	7.3	59.2	--	--	58.9	PS2
A_A Ni	3.1	31.4	33.5	29.3	150	32.9	26.4	22.2	24.2	4.9E9	42.0	1.1	33.1	52.8	12.0	44.4	PS2
X_3A Ni	0.38	23.9	32.1	27.9	21.8	26.1	29.2	25.0	38.6	0.15	14.1	21.5	26.3	35.7	-5.1	25.8	DEM
A_A+3A Ni	1.1	42.7	32.7	24.5	40.3	45.0	29.4	21.2	17.8	25.9	71.6	0.67	44.7	--	--	43.8	PS2

^(a)^—Akaike Information Criterion (AIC). ^(b)^—Bayesian Information Criterion (BIC).

**Table 6:** Adsorption models and fitting parameters for sorption isotherms.

	Langmuir Model				Freundlich Model				Max Exp. *q*_e_ (mg·g^−1^)
	*q*_max_ (mg·g^−1^)	*K*_L_ × 10^3^ (L·mg^−1^)	AIC	BIC	*1/n*_F_	*K*_F_ (mg·g^−1^). (L·mg^-1^)^1/n^	AIC	BIC	
A_A Cu	43.5	2683	28.3	22.5	0.1	22.9	35.2	29.4	47.6
X_3A Cu	105.1 131.0	6.8 236	30.7 (a)	22.5 (a)	0.6 0.1	2.3 35.2	36.8 (a)	28.6 (a)	129.8
A_A+3A Cu	60.4	24.4	32.6	26.8	0.3	8.1	42.6	36.8	55.7
A_A Pb	183.3	116	52.1	46.4	0.3	42.7	61.5	55.7	172.1
X_3A Pb	110.6	52.4	44.2	38.5	0.3	19.9	55.6	49.8	99.9
A_A+3A Pb	346.9	16.9	25.7	17.5	0.7	10.0	28.6	20.5	171.8
A_A Cd	54.0	66.7	41.9	36.1	0.2	14.9	46.6	40.9	51.2
X_3A Cd	102.4	2.9	21.0	12.8	0.7	0.9	23.1	14.9	47.0
A_A+3A Cd	82.9	50.7	40.9	35.2	0.3	17.1	43.5	37.8	81.9
A_A Ni	68.2	34.6	41.4	35.6	0.3	10.5	32.2	26.4	68.2
X_3A Ni	69.2	4.2	22.4	9.8	0.6	1.5	29.2	16.5	42.0
A_A+3A Ni	65.6	21.5	31.0	25.2	0.4	7.7	39.6	33.8	61.3

(a) Values without significance.

**Table 7:** Thermodynamic characterization of the adsorption process for the aerogel A+3A.

Cation	Temperature /°C	*K*_d_ /L·g^−1^	Δ*G*^0^ /kJ·mol^−1^	Δ*H*^0^ /kJ·mol^−1^	Δ*S*^0^ /J·mol^−1^^·^K^−1^	*R* ^2^
Copper	25	0.409	−8.07 ± 0.07	−19 ± 3	−36 ± 9	0.956
	30	0.326	−7.64 ± 0.06			
	35	0.278	−7.4 ± 0.1			
	40	0.267	−7.37 ± 0.02			
	45	0.250	−7.3 ± 0.1			
Lead	25	2.14	−15.10 ± 0.08	225 ± 18	805 ± 58	0.987
	30	17.2	−20.6 ± 0.4			
	35	16.6	−20.9 ± 0.5			
	40	160	−27 ± 1			
	45	859	−32 ± 4			
Cadmium	25	0.080	−5.4 ± 0.2	62 ± 4	226 ± 14	0.985
	30	0.124	−6.6 ± 0.1			
	35	0.161	−7.4 ± 0.1			
	40	0.240	−8.6 ± 0.1			
	45	0.410	−10.13 ± 0.00			
Nickel	25	0.238	−6.54 ± 0.08	(a)	(a)	(a)
	30	0.250	−6.8 ± 0.2			
	35	0.232	−6.69 ± 0.04			
	40	0.258	−7.1 ± 0.2			
	45	0.263	−7.24 ± 0.03			

(a) Adsorption performance does not change with temperature—Figure 7.

**Table 8:** Summary of the adjusted condensation/gelation conditions for the synthesis of aerogels and xerogels.

Formulation	[Base]/M	Aging Time/days	Gelation Temperature/°C	Gelation Time
B	1	6	27	2 h
A	1	6	27	10 min
3A	1	1	60	30 min
A+3A	1	6	27	30 min
TRIS	10	6	60	20 min
U	10	6	27	2 h

## Appendix A

To understand the nature of the interactions adsorbent-adsorbate in the sorption process, when its kinetics is described by the double-exponential model, the points corresponding to the first step were fitted using a surface reaction model—pseudo-second order, and a diffusion describing model—intraparticle diffusion. The latter is presented in Equation (A1), where *k* is the rate constant and *C* is a constant. The result of this analysis is compiled in Table A1:

(A1) $$q_{t}=kt^{0.5}+C$$

**Table A1:** Parameters of kinetic models for the fast step of the kinetic curve.

	Intraparticle Diffusion Model				Pseudo-Second Order			
	*k* (mg·g^−1^^·^h^−0.5^)	*C* (mg·g^−1^)	AIC ^(a)^	BIC ^(b)^	*k*_2_ × 10^3^ (g·mg^−1^^·^h^−1^)	*q*_e_ (mg·g^−1^)	AIC ^(a)^	BIC ^(b)^
A_A Cu	13.4	11.1	39.3	26.7	4767	26.4	25.7	13.1
X_3A Cu	10.1	3.3	28.3	15.7	997.3	13.5	12.8	0.1
X_3A Pb	41.3	6.3	45.9	20.7	566.6	36.6	26.4	1.2
A_A Cd	17.4	3.8	29.1	16.4	455.5	21.7	26.4	13.7
X_3A Ni	8.0	2.3	34.5	9.4	1461	10.5	15.0	−10.2

^(a)^—Akaike Information Criterion (AIC). ^(b)^—Bayesian Information Criterion (BIC).

The results of Table A1 clearly show that the kinetic of adsorption in the fast step is not limited by diffusion, with the PS2 model fitting the data satisfyingly. Hence, it can be said that chemisorption is the limiting phenomena for the adsorption process in this step and the observed kinetic curve is due to the existence of multiple types of active sites, with different accessibilities and surface reaction speeds.
